# Metabolomic exploration of the effects of habituation to livestock trailer and extended transportation in goats

**DOI:** 10.3389/fmolb.2022.1027069

**Published:** 2022-11-17

**Authors:** Phaneendra Batchu, Aditya Naldurtiker, Brou Kouakou, Thomas H. Terrill, George W. McCommon, Govind Kannan

**Affiliations:** Agricultural Research Station, Fort Valley State University, Fort Valley, GA, United States

**Keywords:** goats, habituation, plasma metabolomics, stress, transportation

## Abstract

Goats raised for meat production are often transported long distances. Twelve-month-old male Spanish goats were used to determine the effects of habituation to trailers on plasma metabolomic profiles when transported for extended periods. In a split-plot design, 168 goats were separated into two treatment (TRT; whole plot) groups and maintained on two different paddocks. Concentrate supplement was fed to one group inside two livestock trailers (habituated group, H), while the other group received the same quantity of concentrate, but not inside the trailers (non-habituated, NH). Goats were subjected to a 10-h transportation stress in 4 replicates (*n* = 21 goats/replicate/TRT) after 4 weeks of habituation period. Blood samples were collected prior to loading, 20 min after loading (0 h), and at 2, 4, 6, 8, and 10 h of transportation (Time; subplot). A targeted quantitative metabolomics approach was employed to analyze the samples. The data were analyzed using R software and MIXED procedures in SAS. Several amino acids (alanine, serine, glycine, histidine, glutamate, trans-hydroxyproline, asparagine, threonine, methylhistidine, ornithine, proline, leucine, tryptophan) were higher (*p* < 0.05) in the H group compared to the NH group. Six long-chain acylcarnitines were higher (*p* < 0.05), while free (C0) and short-chain (C3, C5) carnitines were lower (*p* < 0.05) in the NH goats compared to the H goats. In general, amino acid concentrations decreased and long-chain acylcarnitine (>C10) levels increased with transportation time (*p* < 0.05). Butyric acid, α-ketoglutaric acid, and α-aminoadipic acid concentrations were lower (*p* < 0.05) and β-hydroxybutyric acid concentrations were higher in the NH goats compared to the H goats. Plasma glucose, non-esterified fatty acid (NEFA) and urea nitrogen concentrations were significantly influenced by Time (*p* < 0.01). Plasma NEFA concentrations were significantly lower (*p* < 0.01) in the H group than the NH group. Habituation to trailers can be beneficial in enhancing stress coping abilities in goats due to higher concentrations of metabolites such as butyrate and certain amino acids that support antioxidant activities and immune function. Plasma long-chain acylcarnitines may be good indicators of stress during long-distance transportation in goats.

## Introduction

In the US, goats raised for meat production are often transported long distances under commercial situations. Long-distance transportation results in physiological changes in animals to maintain body homeostasis, and adverse effects of stress become evident when physiological mechanisms fail to counterbalance. Assuring the well-being of animals during transportation is becoming a growing societal concern since the negative effects of stress can be prolonged for days after transportation.

Food animals are exposed to various stress factors, such as handling, loading and unloading, novel environment, noise, motion, and vibration, disruption of social structure, food and water deprivation, and extreme temperature and humidity conditions (Kannan et al., 2000; Minka and Ayo, 2009). Severe preslaughter stress related to transportation has been reported as one of the major factors affecting meat quality in small ruminants (Kannan et al., 2003). Evidence from previous studies suggests that transportation stress elicits metabolic changes that impact adrenocortical activity, energy balance, immune response, and body weight in goats (Kannan et al., 2000).

To evaluate stress in food animals, researchers have used a wide range of physiological indicators, and studies have also focused on the validity of these animal welfare indices (Verbeke and Viaene, 2000). Increase in plasma non-esterified fatty acid (NEFA) concentrations have been observed in goats after feed deprivation and transportation (Kannan et al., 2002, 2003) and in sheep after 8 h of transportation (Zhong et al., 2011). Blood glucose and urea nitrogen (BUN) concentrations increase due to transportation in goats and remain elevated after transportation during the initial hours of holding (Kannan et al., 2000). Stress due to heat and transportation causes higher β-hydroxybutyrate concentrations in goats and other ruminants (Salama et al., 2014; Batchu et al., 2021).

The metabolome is a collection of small molecular mass components found in biological media, and metabolomic analysis involves large-scale detection and quantification of metabolites (Junot et al., 2014). Advanced analytical techniques and chemometrics are used to identify a vast number of metabolites in a sample, including amino acids, sugars, ketones, fatty acids, organic acids, and exogenous small molecules. Our previous study indicated that stress has a significant impact on the plasma metabolome in goats, with the amino acid levels decreasing and medium- and long-chain acylcarnitine concentrations increasing with increasing duration of stress (Batchu et al., 2021). Therefore, plasma acylcarnitine concentrations could reflect oxidation rate of fatty acids and amino acids in tissues, particularly in liver and muscle (Xu et al., 2011).

The behavioral and physiological responses of an animal can be negatively affected when exposed to a novel situation, and repeated exposure to the same stressor such as handling can attenuate these responses (Ujita et al., 2021). Habituating to transportation has been reported to significantly decrease the frequencies of behaviors indicative of stress as well as physiological stress responses in donkeys (Dai et al., 2020). These authors further observed that habituation to transportation reduced the time needed to load donkeys onto the vehicle. Weeks et al. (2012) also reported that regardless of the age of horses, habituation made the loading process considerably easier. Habituating animals to transportation may help animals cope with the detrimental effects of stress (Stockman et al., 2011); however, to what extent this can be applied in commercial conditions is questionable. We propose habituating goats to livestock trailers may be a more practicable method that could be easily adopted by goat producers worldwide, who invariably operate on smaller-scale and with limited resources.

There are no data available on the effects of conditioning goats to livestock trailers on stress responses during transportation. Recent studies conducted in other livestock species have confirmed the positive effects of habituation to handing and transportation in reducing stress-related physiological responses (Dai et al., 2020; Ujita et al., 2021). The objective of this study was to determine the effects of habituation to livestock trailers on plasma metabolomic profiles in goats.

## Materials and methods

### Animals

The protocol for this research was reviewed and approved by Fort Valley State University’s Animal Care and Use Committee prior to beginning the experiment. Twelve-month-old male Spanish goats were used to determine the effects of habituation to trailers on plasma metabolomic profiles when transported for long periods. The goats were dewormed 3 weeks before the study. All animals were examined for general health status and were determined to be healthy prior to beginning of the experiment. In a split-plot design, 168 uncastrated male Spanish goats (12-month old; Average BW = 31.6 ± 0.34 kg) were separated into two treatment (TRT; whole plot) groups and maintained on two different grass paddocks (predominantly Bermudagrass, *Cyanodon* *dactylon*). Concentrate supplement (commercial goat pellet, 14% crude protein) was fed to one group inside two livestock trailers (5.3 × 2.3 m each; habituated group, H), while the other group received the same quantity of concentrate, but not inside the trailers (non-habituated, NH). Habituation to trailer was conducted during feeding time between 8:30 a.m. and 9:30 a.m. every day during the months of March-April. The average high/low temperatures in March were 21.1°C/7.2°C and in April were 25.0°C/10.6°C. Every day, the animals remained in the trailer for a 50 ± 10 min-period until all the concentrate feed was consumed. Goats were subjected to a 10-h transportation stress on 4 consecutive days (replicates; *n* = 21 goats/replicate/TRT) after 4 weeks of habituation period. The livestock trailers used for habituation and transportation were identical in dimensions and positions of windows provided for ventilation. The average temperatures on the days 1, 2, 3, and 4 of transportation trials were 17.8, 21.7, 22.5, and 23.1°C, respectively. The average relative humidity percentages were 68.0, 73.5, 68.5, and 79.0, respectively, on days 1, 2, 3, and 4. Each trailer was partitioned into two compartments with H goats in one compartment and the NH goats in the other. The order of loading of goats onto the trailer was alternated on each day, such that H goats were in the front compartment on 1 day and they were in the rear compartment on the next day. The floor space allocated was 0.29 m^2^/animal during transportation in all replicates that allowed adequate air circulation. The goats were transported approximately 550 km at an average speed of 61 km/h with a 10-min stop every 2 h for blood sampling. To be consistent and to minimize vehicular vibrations, the same route that comprised of paved roads was followed during transportation on all 4 days.

### Blood sampling

Blood samples were collected prior to loading (Preload, PL), 20 min after loading (0 h), and at 2, 4, 6, 8, and 10 h of transportation (Time; subplot). For 2, 4, and 8 h sampling, the truck was stopped for 10 min at each time period and blood samples were collected inside the trailer to avoid repeated unloading and loading animals. Only two individuals had to enter the trailer, one animal handler and one blood sampler, for blood sampling. All efforts were made not to agitate the goats, including avoiding loud noise and rough handling. After blood sampling, each goat was marked on the horns with a colored marker to avoid being sampled again. Blood samples were collected by a trained individual by jugular venipuncture into K_2_EDTA-coated vacutainer tubes and kept on ice until separation of plasma. Blood samples were collected without any time lapse after the goats were caught in order to avoid confounding of the effect of blood sampling. The individual who collected the blood samples at all time points was so proficient such that it took only a few seconds (<30 s) to draw a sample from each animal. The tubes were then centrifuged at 1,000 × g for 20 min for separation of plasma. Plasma samples were pipetted into screw-cap vials and stored at −80°C until analysis. For blood glucose, BUN, and creatine concentrations, samples were obtained separately in 3 ml vacutainer tubes coated with EDTA (K3) and kept on ice until analysis.

### Plasma NEFA, BUN, creatine, and glucose concentrations

The NEFA-HR (2) Kit (Fujifilm, Mountain View, CA) was used to determine plasma NEFA concentrations. The colorimetric assay was conducted using 96-well micro-titer plates according to the instructions provided by the manufacturer. Briefly, plasma samples (5 μL) were placed in the wells, followed by 200 μL of color reagent A solution. The plates were then incubated for 5 min at 37°C before the first optical density measurement was made using a microplate reader at a wavelength of 550 nm (Synergy HTX Microplate Reader, Bio-Tek, Winooski, VT). Then, 100 μL of color reagent B solution was added to each well, and the optical density was measured again at 550 nm. By measuring against a standard curve generated using the manufacturer’s instructions and following the manufacturer’s directions, the difference between the optical density readings was used to estimate NEFA concentrations in each sample. The concentrations of glucose, BUN, and creatine were determined using the VETSCAN HM5 Hematology Analyzer (Abaxis, Union City, CA) according to the manufacturer’s protocol.

### Plasma metabolomics

All 168 plasma samples (*n* = 21 goats/replicate/TRT) were shipped on dry ice to The Metabolomics Innovation Center (TMIC) at the University of Alberta, Edmonton, Canada for metabolomics analysis. The samples were analyzed utilizing a targeted quantitative metabolomics technique that combined direct injection mass spectrometry with a reverse-phase LC–MS/MS custom assay. This custom assay, in combination with a mass spectrometer, was used to identify and quantify up to 150 different endogenous metabolites, such as amino acids, acylcarnitines, biogenic amines and derivatives, uremic toxins, glycerophospholipids, sphingolipids, and sugars. Derivatization and extraction of analytes were combined with selective mass-spectrometric detection using multiple reaction monitoring (MRM) pairs in this approach.

Samples were thawed on ice, vortexed and centrifuged at 13,000 × g for all metabolites except organic acids. The center of the filter on the upper 96-well plate was loaded with 10 µL of each sample, which was then dried in a nitrogen stream. Then, phenyl-isothiocyanate was added for derivatization. The filter spots were dried again with an evaporator after incubation. The metabolites were extracted using 300 µL of extraction solvent. Centrifugation into the lower 96-deep well plate yielded the extracts, which were then diluted with MS running solvent.

For organic acid analysis, 50 µL of sample was mixed with 150 µL of ice-cold methanol and 10 L isotope-labeled internal standard mixture for overnight protein precipitation. It was then centrifuged for 20 min at 13000 × g. A 96-deep well plate was loaded with 50 µL of supernatant, followed by the addition of 3-nitrophenylhydrazine (NPH) reagent. Before LC-MS injection, BHT stabilizer and water were added after a 2-h incubation.

An ABSciex 4000 Qtrap^®^ tandem mass spectrometry instrument (Applied Biosystems/MDS Analytical Technologies, Foster City, CA) with an Agilent 1260 series UHPLC system (Agilent Technologies, Palo Alto, CA) was used for mass spectrometric analysis. An LC approach was used to deliver the samples to the mass spectrometer, followed by a direct injection (DI) method. Analyst 1.6.2 was used to analyze the data.

### Statistical analysis

Plasma glucose, NEFA, BUN, and creatine concentration data were analyzed using MIXED procedures in SAS. When significant by ANOVA, the means were separated using the pdiff procedure.

Data from all 168 samples were used for metabolomics analysis. The metabolites with identical concentrations for all samples (ex. 0 µM) and those with more than 20% of missing concentrations were removed from the datasets. For multivariate analysis, data were scaled by range scaling with Metaboanalyst R. Samples with missing time points were removed and the data were log-transformed for analysis of variance. For two-group comparisons, the data from different time points were combined and univariate T-test and effect size calculation were performed for each metabolite. Because different sets of animals were used at different time points, one-way ANOVA tests were performed for longitudinal analysis of time points, followed by post-hoc tests and effect size calculations. For comparison of TRT (H vs. NH) at different time points, two-way ANOVA was conducted. For all types of comparisons, PCA and PLS-DA tests were performed.

Since the data for all groups were not normally distributed, univariate analysis was conducted using a non-parametric version of statistical tests. Specifically, T test for two independent samples was conducted with Mann-Whitney U rank method. The effect size was calculated with the Cliff’s Delta method. Fold change was determined by calculating the ratio between group medians. One-way ANOVA was performed using Kruskal–Wallis test. ANOVA post-hoc tests were conducted using the Dunn’s test with Benjamini Hochberg False Discovery Rate correction for multiple comparisons. The effect size was calculated with the Cliff’s Delta method (Vargha and Delaney, 2000; Macbeth et al., 2011). Fold change was determined by calculating the ratio between group medians. Two-way ANOVA and post-hoc tests were conducted on log-transformed data, using Benjamini Hochberg False Discovery Rate method to correct *p*-values for multiple comparisons. To balance the risk of Type 1 and Type 2 errors, thresholds of 0.05 for raw *p*-values and 0.1 for FDR values were used to identify statistically significant changes in metabolite concentrations from the T test.

Metaboanalyst R was used to perform principal component analysis (PCA) and partial least square discriminant analysis (PLS-DA). The PLS-DA, a multivariate supervised pattern recognition method, maximizes discriminating variation between classes. The models were tested for performance and the absence of overtraining with 10-fold cross-validation. The model accuracy was considered satisfactory when R2 and Q2 were above 0.66 and considered not over-trained when R2 and Q2 were comparable with each other (within 20%). A permutation test was conducted to assess statistical significance of PLS-DA model. A model was considered statistically significant if *p* < 0.05. The metabolites were then plotted according to their importance in separating the different treatment groups and transportation time groups based on the PLS-DA results using variable importance in projection (VIP) scores. A VIP score of >1.0 indicates that the metabolite is significantly involved in separation of the classes.

Day (replicate) effects were removed by the commonly used median batch effect correction (Rusilowicz et al., 2016). Median concentrations of metabolites were calculated and a batch with the largest median value was selected as the reference per metabolite. For the remaining batches, correction factors for each metabolite were calculated by subtracting the batch median value from the reference median value. Finally, concentrations of each metabolite in the remaining batches were adjusted by adding the corresponding correction factors.

## Results

**FIGURE 1:**
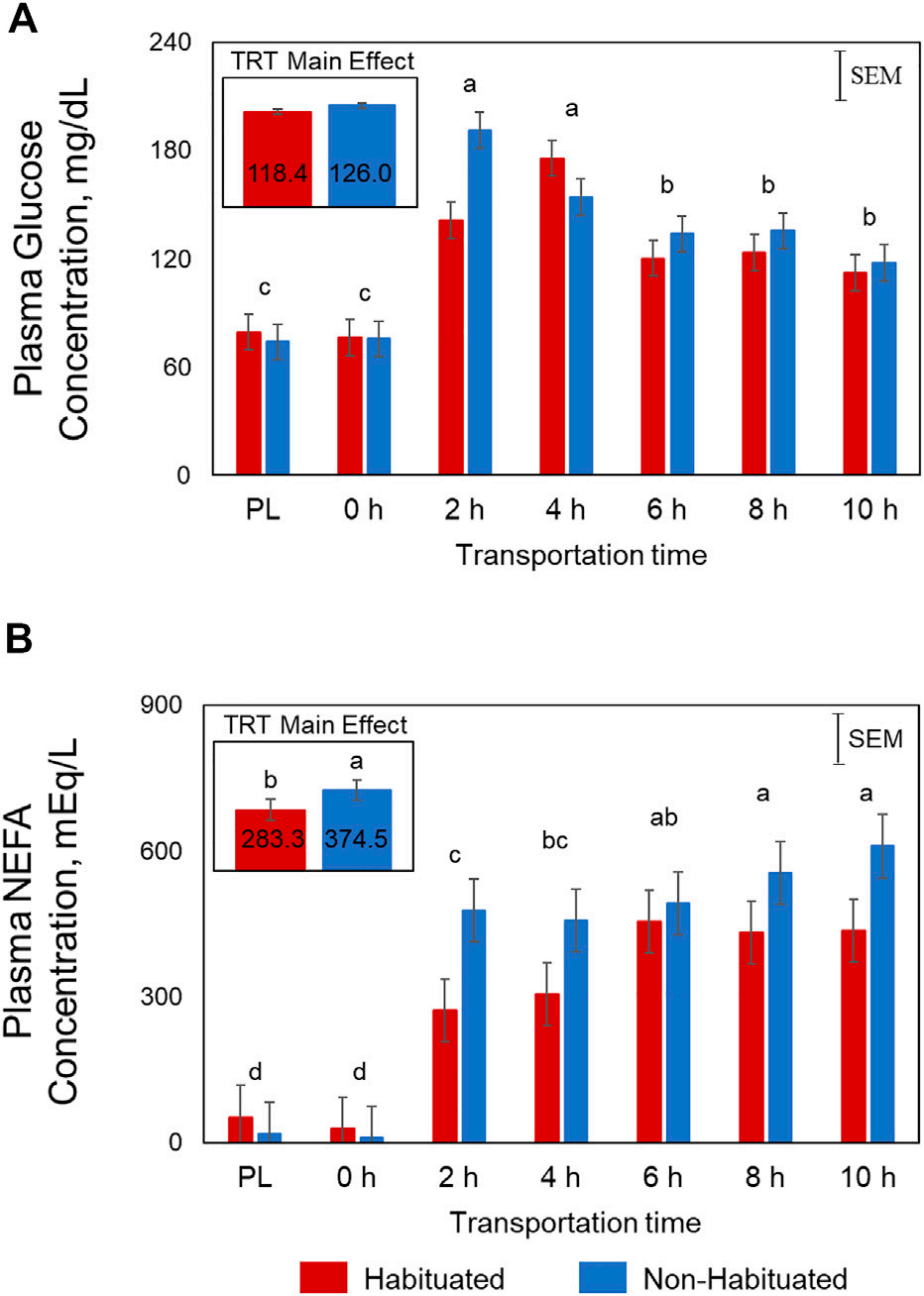
Effects of habituation treatment (TRT) and transportation time (Time; PL = Preload) on plasma **(A)** glucose (TRT, *p* = 0.14; Time, *p* < 0.01; TRT × Time, *p* < 0.05) and **(B)** non-esterified fatty acid (NEFA; TRT, *p* < 0.05; Time, *p* < 0.05) concentrations in goats.

**FIGURE 2:**
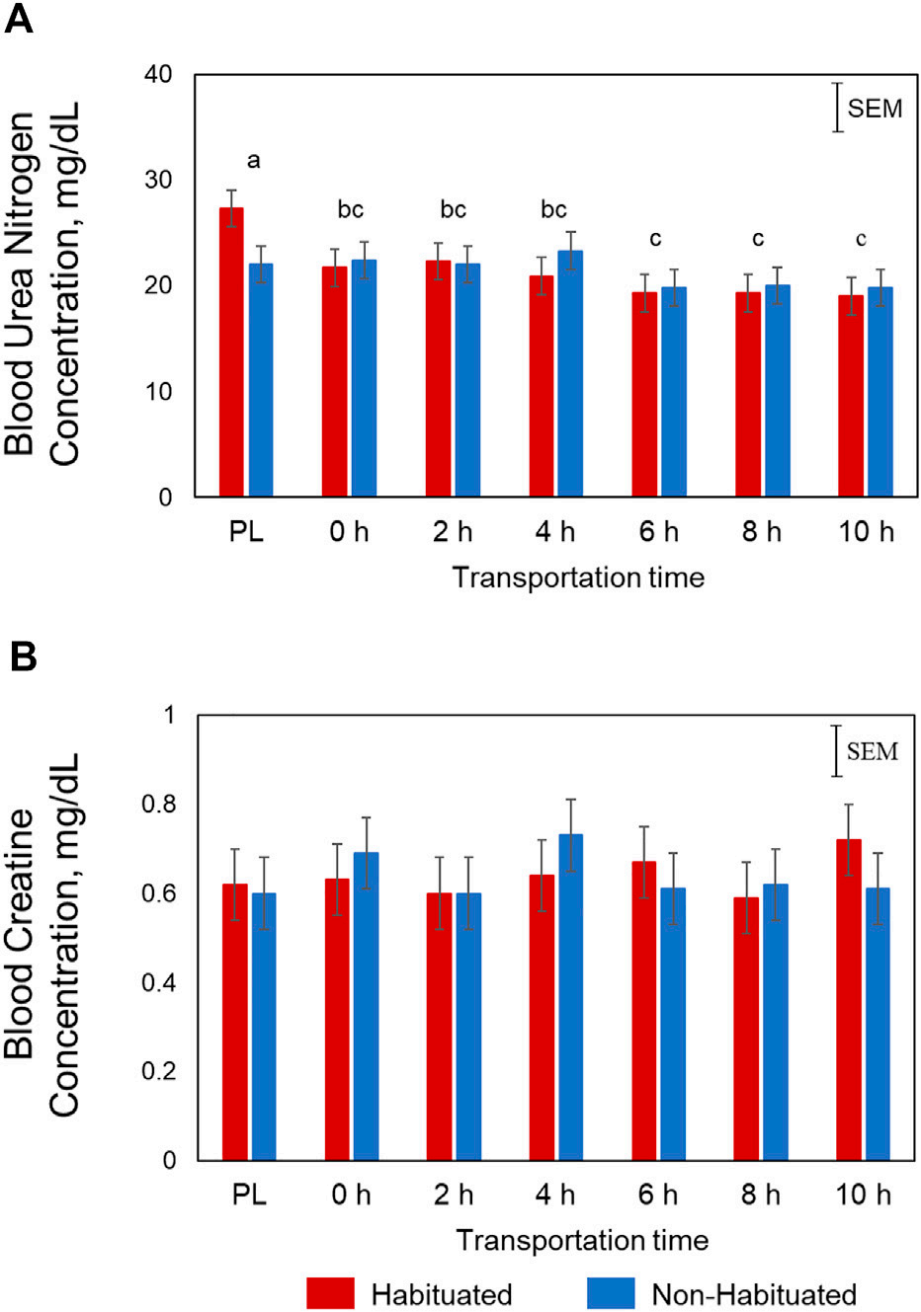
Effects of habituation treatment (TRT) and transportation time (Time; PL = Preload) on plasma **(A)** urea nitrogen (Time, *p* < 0.05), and **(B)** creatine concentrations in goats.

Plasma glucose concentrations were significantly influenced by Time (*p* < 0.01) and TRT × Time (*p* < 0.05; Figure 1A). In the NH goats, glucose concentrations spiked at 2 h before gradually decreasing, yet remained higher than PL and 0 h levels. However, in the H group, the glucose concentrations increased gradually and peaked at 4 h. The Time main effects showed that the concentrations were highest at 2 and 4 h, lowest at PL and 0 h sampling, and intermediate at 6, 8, and 10 h for both groups. Plasma NEFA concentrations were significantly higher (*p* < 0.05; Figure 1B) in the NH group compared to the H group. Plasma NEFA concentrations were low at PL and 0 h sampling, significantly increased at 2 h, and further increased with increasing transportation time (*p* < 0.05) in both groups (Time main effect). The overall BUN concentrations were high at PL sampling, low at 6, 8, and 10 h, and intermediate at 0, 2, and 4 h (*p* < 0.05; Figure 2A). Plasma creatine concentrations were not affected by any of the factors (Figure 2B).

At the metabolome level, 13 amino acids, 12 acylcarnitines, 25 phosphatidylcholines, and sphingomyelins, and 13 other metabolites were significantly affected (*p* < 0.05) by TRT. Of the 13 amino acids, 12 (alanine, serine, glycine, tryptophan, histidine, glutamic acid, trans-hydroxyproline, asparagine, threonine, ornithine, proline, and leucine) were significantly lower in the NH group, while methylhistidine was higher in the NH group compared to the H group (Table 1). Eight of the acylcarnitines were higher in the NH group and 4 were lower in the NH group compared to the H group (Table 1). All 25 phosphatidylcholines and sphingomyelins that were significantly influenced by TRT were higher in the NH group compared to the H group (Table 2). In addition, HPHPA, β-hydroxybutyrate, creatinine, and acetyl-ornithine concentrations were higher in the NH groups, while methylmalonic acid, kynurenine, indole acetic acid, α-ketoglutaric acid, propionic acid, uric acid, putrescine, butyric acid, and α-aminoadipic acid were lower in the NH group compared to the H group (Table 3).

**TABLE 1:** Amino acids and acylcarnitines significantly (*p* < 0.05) affected by treatment (H = Habituated; NH = Non-habituated) in goats.

Metabolite	*p*-value	FDR^a^	Fold change	Cliff’s delta effect size	Cliff’s delta effect level	Direction of change
Amino Acids						
Alanine	9.31e-05	2.04e-03	0.85	0.33	Medium	↓ in NH
Serine	1.12e-04	2.04e-03	0.81	0.33	Small	↓ in NH
Glycine	1.42e-04	2.04e-03	0.90	0.32	Small	↓ in NH
Tryptophan	4.90e-04	4.24e-03	0.86	0.29	Small	↓ in NH
Histidine	1.13e-03	8.11e-03	0.91	0.27	Small	↓ in NH
Glutamic acid	0.013	0.043	0.85	0.20	Small	↓ in NH
trans-Hydroxyproline	0.013	0.044	0.92	0.20	Small	↓ in NH
Asparagine	0.017	0.054	0.79	0.19	Small	↓ in NH
Threonine	0.021	0.062	0.85	0.18	Small	↓ in NH
Methylhistidine	0.027	0.075	1.04	−0.17	Small	↑ in NH
Ornithine	0.032	0.078	0.91	0.17	Small	↓ in NH
Proline	0.036	0.085	0.91	0.16	Small	↓ in NH
Leucine	0.039	0.086	0.93	0.16	Small	↓ in NH
Acylcarnitines						
Octadecanoylcarnitine (C18)	1.40e-05	5.80e-04	1.22	−0.37	Medium	↑ in NH
Propionylcarnitine (C3)	4.77e-04	4.24e-03	0.88	0.30	Small	↓ in NH
Dodecanedioylcarnitine (C12DC)	4.82e-04	4.24e-03	0.89	0.30	Small	↓ in NH
Acetylcarnitine (C2)	7.36e-04	5.58e-03	1.17	−0.28	Small	↑ in NH
Hexadecanoylcarnitine (C16)	1.71e-03	0.011	1.15	−0.26	Small	↑ in NH
Tetradecenoylcarnitine (C14:1)	1.79e-03	0.011	1.14	−0.26	Small	↑ in NH
Octadecenoylcarnitine (C18:1)	4.54e-03	0.020	1.14	−0.23	Small	↑ in NH
Isovalerylcarnitine (C5)	0.016	0.050	0.97	0.19	Small	↓ in NH
Hydroxyisovalerylcarnitine (C5OH)	0.024	0.067	0.96	0.18	Small	↓ in NH
Tetradecanoylcarnitine (C14)	0.038	0.086	1.07	−0.16	Small	↑ in NH
Decanoylcarnitine (C10)	0.043	0.093	1.07	−0.15	Small	↑ in NH
Carnitine (C0)	0.047	0.098	0.95	0.15	Small	↓ in NH

^a^ False Discovery Rate.

**TABLE 2:** Phosphatidylcholines and sphingomyelins significantly (*p* < 0.05) affected by treatment (H = Habituated; NH = Non-habituated) in goats.

Metabolite	*p*-value	FDR^a^	Fold change	Cliff’s delta effect size	Cliff’s delta effect level	Direction of change
Diacylphosphatidylcholine C36:0	1.40e-04	2.04e-03	1.17	−0.32	Small	↑ in NH
Hydroxysphingomyelin C22:1	2.61e-04	3.37e-03	1.16	−0.31	Small	↑ in NH
Sphingomyelin C18:0	4.71e-04	4.24e-03	1.12	−0.30	Small	↑ in NH
Sphingomyelin C18:1	4.93e-04	4.24e-03	1.19	−0.29	Small	↑ in NH
Lysophosphatidylcholine C18:0	5.36e-04	4.33e-03	1.12	−0.29	Small	↑ in NH
Lysophosphatidylcholine C28:1	1.68e-03	0.011	1.15	−0.26	Small	↑ in NH
Diacylphosphatidylcholine C36:6	1.90e-03	0.011	1.08	−0.26	Small	↑ in NH
Diacylphosphatidylcholine C40:1	1.98e-03	0.011	1.08	−0.26	Small	↑ in NH
Lysophosphatidylcholine C18:2	2.12e-03	0.011	1.18	−0.26	Small	↑ in NH
Diacylphosphatidylcholine C32:2	2.71e-03	0.014	1.09	−0.25	Small	↑ in NH
Lysophosphatidylcholine C26:1	2.77e-03	0.014	1.12	−0.25	Small	↑ in NH
Diacylphosphatidylcholine C38:0	3.11e-03	0.015	1.22	−0.24	Small	↑ in NH
Hydroxysphingomyelin C24:1	4.50e-03	0.020	1.06	−0.23	Small	↑ in NH
Lysophosphatidylcholine C26:0	7.32e-03	0.029	1.13	−0.22	Small	↑ in NH
Acyl alkylphosphatidylcholine C36:0	7.39e-03	0.029	1.12	−0.22	Small	↑ in NH
Sphingomyelin C16:0	8.64e-03	0.033	1.09	−0.21	Small	↑ in NH
Sphingomyelin C16:1	9.74e-03	0.036	1.10	−0.21	Small	↑ in NH
Lysophosphatidylcholine C24:0	0.011	0.039	1.08	−0.20	Small	↑ in NH
Hydroxysphingomyelin C16:1	0.021	0.062	1.09	−0.18	Small	↑ in NH
Lysophosphatidylcholine C20:4	0.023	0.067	1.08	−0.18	Small	↑ in NH
Lysophosphatidylcholine C18:1	0.028	0.075	1.07	−0.17	Small	↑ in NH
Hydroxysphingomyelin C14:1	0.030	0.077	1.06	−0.17	Small	↑ in NH
Sphingomyelin C20:2	0.031	0.078	1.07	−0.17	Small	↑ in NH
Lysophosphatidylcholine C16:0	0.044	0.094	1.03	−0.15	Small	↑ in NH
Lysophosphatidylcholine C28:0	0.047	0.098	1.07	−0.15	Small	↑ in NH

^a^ False Discovery Rate.

**TABLE 3:** Metabolites significantly (*p* < 0.05) affected by treatment (H = Habituated; NH = Non-habituated) in goats.

Metabolite	*p*-value	FDR^a^	Fold change	Cliff’s delta effect size	Cliff’s delta effect level	Direction of change
HPHPA	7.00e-07	9.03e-05	1.32	−0.43	Medium	↑ in NH
Methylmalonic acid	6.22e-06	4.01e-04	0.84	0.39	Medium	↓ in NH
β-Hydroxybutyric acid	1.80e-05	5.80e-04	1.24	−0.37	Medium	↑ in NH
Kynurenine	1.26e-04	2.04e-03	0.77	0.33	Small	↓ in NH
Indole acetic acid	6.33e-03	0.027	0.75	0.22	Small	↓ in NH
α-Ketoglutaric acid	6.64e-03	0.028	0.96	0.22	Small	↓ in NH
Propionic acid	0.011	0.038	0.91	0.21	Small	↓ in NH
Uric acid	0.015	0.050	0.91	0.19	Small	↓ in NH
Putrescine	0.029	0.076	0.94	0.17	Small	↓ in NH
Butyric acid	0.032	0.078	0.95	0.17	Small	↓ in NH
Creatinine	0.036	0.085	1.05	−0.16	Small	↑ in NH
α-Aminoadipic acid	0.038	0.086	0.92	0.16	Small	↓ in NH
Acetyl-ornithine	0.048	0.098	1.04	−0.15	Small	↑ in NH

^a^ False Discovery Rate.

**FIGURE 3:**
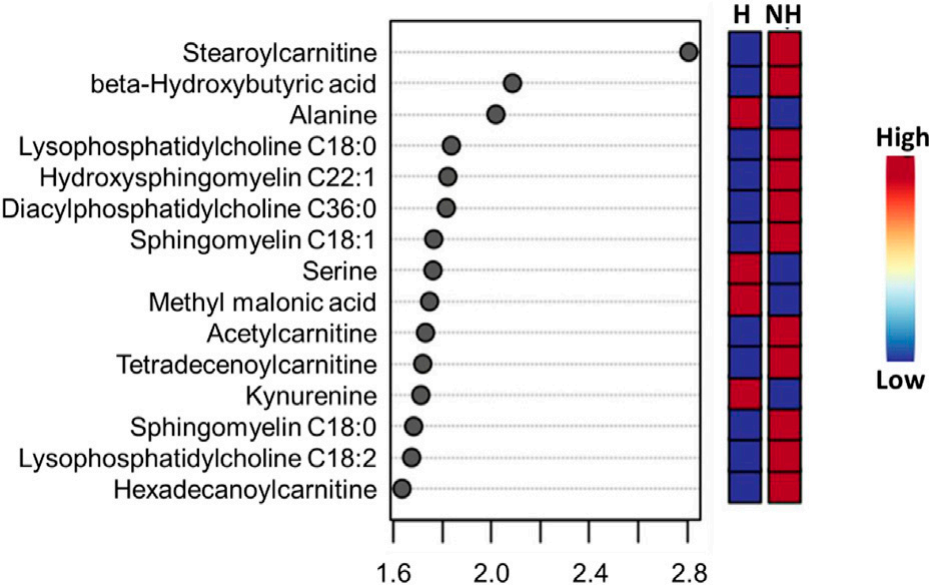
PLS-DA VIP plot showing differences between treatment (H = Habituated; NH = Non-habituated) groups and the metabolites (VIP scores >1.5) that significantly contribute to the difference. The metabolite concentrations averaged across all time points were used in the PLS-DA model (*p* < 0.05).

Visualization of metabolites clustered by means of a heatmap also revealed that the majority of the amino acids were higher in the H group, while most of the phoshatidylcholines and sphingomyelins and acylcarnitines were lower in the H group compared to the NH group, as evidenced by the intensity of red color (Supplementary Figure S1). When averaged across all time points, stearoylcarnitine, β-hydroxybutyric acid, alanine, lysophosphatidylcholine C18:0, hydroxysphingomyelin C22:1, diacylphasphotidylcholine C36:0, sphingomyelin C18:1, serine, methyl malonic acid, acetylcarnitine, kynurenine, sphingomyelin C18:0, lysophosphatidylcholine C18:2, and hexadecanoylcarnitine were the top 15 metabolites identified by PLS-DA multivariate model (*p* < 0.05) and VIP values with the highest influence (VIP scores >1.5) in separating the H and NH groups, as shown in Figure 3.

**FIGURE 4:**
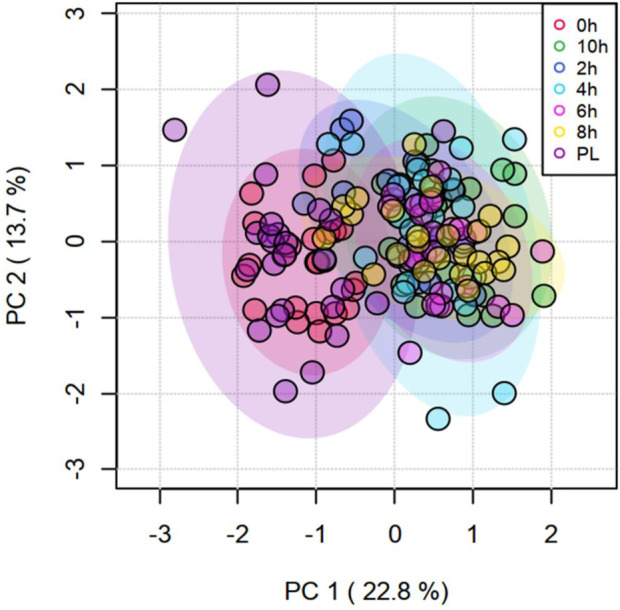
Principal Component Analysis (PCA) plot of principal components 1 and 2 of transportation time for metabolites.

**FIGURE 5:**
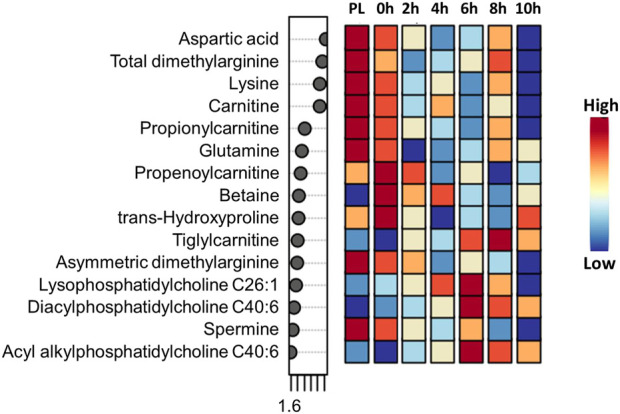
PLS-DA VIP plot showing differences among transportation time (PL = Preload) groups and the metabolites (VIP scores >1.5) that significantly contribute to the difference. The metabolite concentrations averaged across the two treatments were used in the PLS-DA model (*p* < 0.05).

Time had a significant effect (*p* < 0.05) on 20 amino acids, with the concentrations decreasing with increasing transportation time as shown in the box plots (Supplementary Figure S2). Several long-chained acylcarnitine concentrations increased (*p* < 0.05; Supplementary Figure S3) with increasing transportation time, while this pattern was not apparent with short-chained acyl carnitines. In addition, carnosine, pyruvic acid, α-amino adipic acid, butyric acid, hippuric acid, lactic acid, kynurenine, indole acetic acid, spermine, citric acid, serotonin, and HPHPA were also significantly (*p* < 0.05) affected by Time (Supplementary Figure S4). The changes in concentrations of all metabolites that were significantly influenced by Time are shown using a heatmap (Supplementary Figure S5). The PCA plot created to visualize the separation of metabolites by Time in principal components 1 and 2 showed that the clusters corresponding to different time periods overlapped; however, the clusters representing PL and 0 h spaced slightly apart from clusters of other time periods (Figure 4). The top 15 metabolites identified by PLS-DA multivariate model (*p* < 0.05) and VIP values are shown in Figure 5. Aspartic acid, total dimethylarginine, lysine, carnitine, propionylcarnitine, glutamine, propenoylcarnitine, betaine, trans-hydroxyproline, tiglylcarnitine, asymmetric dimethylarginine, lysophosphatidylcholine C26:1, diacylphosphatidylcholine C40:6, spermine, and acyl alkylphosphatidylcholine C36:0 had the highest influence (VIP scores >1.5) in separating the different time periods.

**FIGURE 6:**
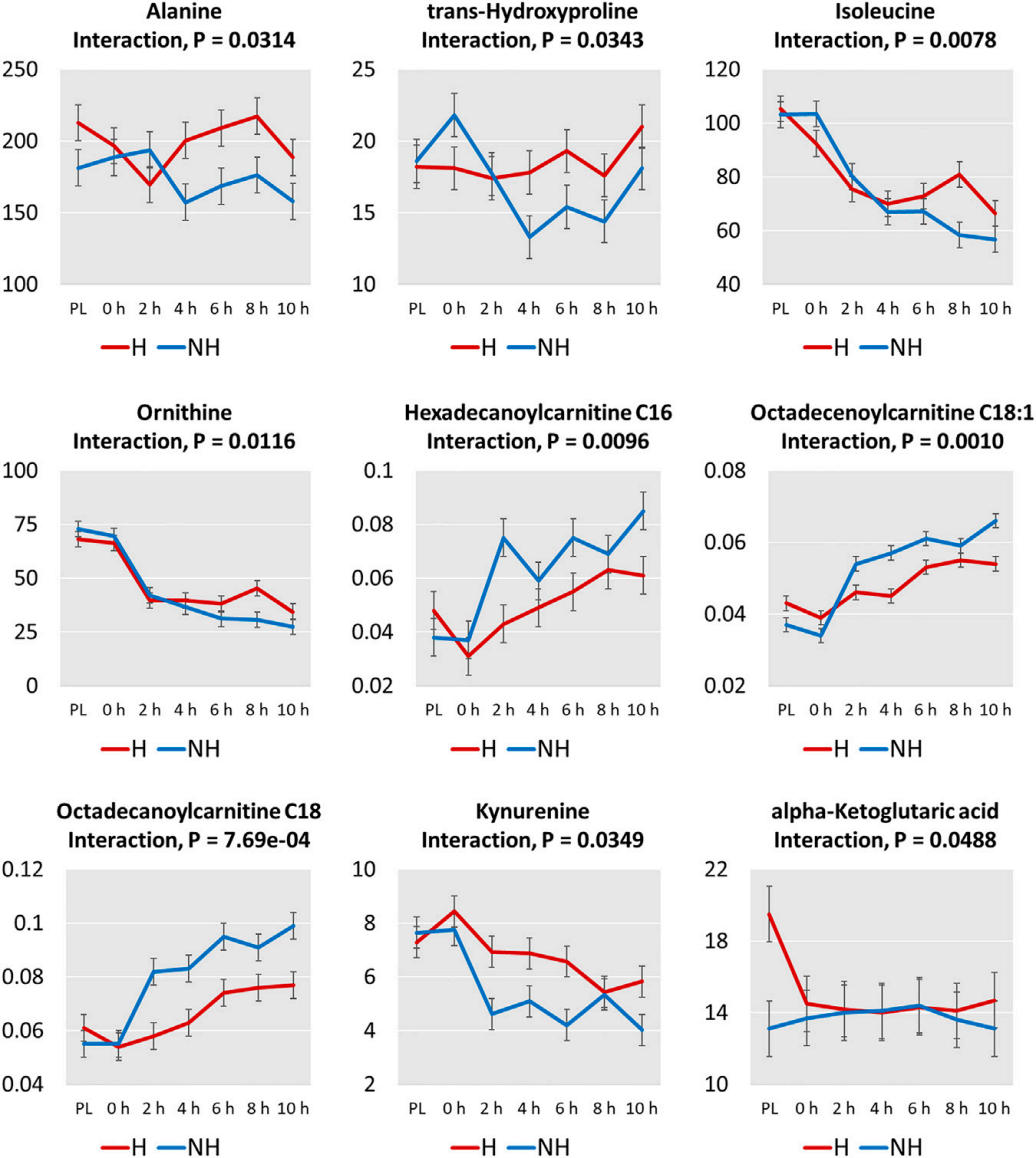
Plots of means (± SEM) of metabolites with significant treatment (H = Habituated; NH = Non-habituated) × transportation time (PL = Preload) interaction effects (*p* < 0.05).

Interaction effects (TRT × Time) were significant (*p* < 0.05; Figure 6) for α-ketoglutaric acid, kynurenine, 4 amino acids (alanine, trans-hydroxyproline, isoleucine, ornithine), and 3 acylcarnitines (hexadecanoylcarnitine, octadecenoylcarnitine, octadecanoylcarnitine). The increases in concentrations of the 3 acylcarnitines over transportation time was greater in the NH group compared to the H group. The concentrations of the 3 amino acids decreased to a greater extent after 10 h in the NH group compared to the H group. The α-ketoglutaric acid concentration at PL sampling was significantly higher (*p* < 0.05) in the H group compared to the NH group but decreased rapidly at 0 h and remained at that level throughout the transport duration. However, α-ketoglutaric acid concentrations remained low at all sampling periods.

## Discussion

### Stress indicators

When an animal is exposed to a stressor, catecholamine release from adrenal medulla causes glycogenolysis in the liver and lipolysis of adipose tissue that lead to increase in both blood glucose and NEFA concentrations (Kannan et a., 2000; 2002; Saeb et al., 2010). Adipocytes are lipase sensitive during stress, and the stored triacylglycerol is split into glycerol and NEFA. Changes in blood glucose concentrations result in glucocorticoid release that stimulates liver to convert fat and protein to intermediary metabolites that are used for energy production (Saeb et al., 2010). Gluconeogenic substrates, such as amino acids and short-chain fatty acids, also decrease during prolonged energy demand and deficit. As a result, fatty acids may be the main source of energy during these situations, and the primary fatty acid metabolic organ is the liver.

Plasma NEFA concentrations were significantly lower in the H group compared to NH group as shown in Figure 1. These effects suggest that habituation to livestock trailer was efficient in reducing stress responses in goats during long-distance transportation. Mobilization of fatty acids from adipose tissue and higher NEFA blood concentrations may provide energy for the animals. Blood urea nitrogen concentrations decreased with increasing transportation time in both H and NH treatment groups (Figure 2). The BUN concentrations were highest at PL and lowest at 6 h, 8 h and 10 h samplings. Any situation that causes protein catabolism, including elevated blood glucocorticoid concentrations and feed deprivation, will likely increase BUN (Finco, 1997; Kannan et al., 2000). Transport stress causes increase in BUN concentrations in farm animals (Hurtung, 2003) since feed deprivation is confounded with transportation stress and the process invariably increases glucocorticoids (Kannan et al., 2000).

### Amino acids

Several amino acids were lower in the NH group compared to the H group in our study (Table 1). In addition to protein synthesis, amino acids are metabolized to compounds that enter the tricarboxylic acid (TCA) cycle to produce adenosine triphosphate. The amino acids that do not enter the TCA cycle are either ketogenic, glucogenic, or both (Litwack, 2021). Alanine, serine, glycine, histidine, asparagine, proline, and glutamic acid that forms glutamine, are glucogenic amino acids and were probably used for glucose production due to higher stress and energy demand in the NH group. Decreased amino acid concentrations in circulation may indicate their being used in gluconeogenesis, particularly in stressful situations (Coleman et al., 2020). For instance, during feed deprivation, alanine regulates gluconeogenesis to replenish glucose in dairy cattle (Guo et al., 2018). Leucine is a ketogenic amino acid that was used up for fatty acid production due to elevated stress in the NH goats. In addition, tryptophan and threonine were also lower in the NH groups and could have been involved in both glucose and fatty acid formations. Glucose concentrations were not significantly different between the two treatments in our study.

Almost all of the 20 amino acids affected by Time decreased slightly after loading (0 h), further decreased after the beginning of transportation, and stayed at a lower level throughout the rest of the transportation period (Supplementary Figure S2). A progressive decrease with increasing transportation time was noticed only for arginine, ornithine, and isoleucine. Based on interaction effects, the differences in alanine, trans-hydroxyproline, isoleucine, and ornithine concentrations at different time points became significant after 4 h of transportation. While the concentrations of these amino acids decreased or continued to decrease after 4 h in the NH group, the levels increased or stabilized after 4 h in the H group (Figure 6). This may indicate that habituating goats to transportation trailer could result in lower amino acid catabolism.

In addition to their function in protein building, amino acids have several important roles, such as immunomodulatory and immunometabolic activities (Li et al., 2007; Coleman et al., 2020). Glycine and serine concentrations were significantly lower in the NH goats compared to the H goats. Glycine is involved in protein and heme synthesis, as well as in bile acid conjugation (Wang et al., 2013). Its functions also include purine synthesis, glutathione synthesis, cell proliferation and differentiation, and regulating oxidative stress (Chen et al., 2013; Wang et al., 2013). Serine supports glutathione production and has also been reported to support T cell proliferation by supplying glycine and maintaining 1-carbon metabolism (Newsholme et al., 1999; Ma et al., 2017). Histidine that was lower in the NH group plays important roles in immune and antioxidant responses and energy metabolism. In addition, histidine produces glycoproteins that play an important role in immune function-related activities, such as phagocytosis and removal of antibody complexes (Wakabayashi, 2013).

Other amino acids with anti-inflammatory and immune support functions include glutamate and ornithine, whose concentrations were also lower in the NH goats during transportation. Glutamate is a nonessential amino acid and is required for the synthesis of glutathione, NADPH, and α-ketoglutarate (Wu et al., 2004; Newsholme et al., 2003). Glutamic acid converts to glutamine, a purine and pyrimidine precursor, which is required for nitric oxide, cytokine, and NADPH production and accelerated interleukin-6 production by macrophages (Yassad et al., 1997; Newsholme et al., 2003). Ornithine that is synthesized from arginine is required for polyamine synthesis and plays an important role in urea cycle. Arginine concentrations sharply decreased after beginning of transportation and stayed at low levels throughout the transportation period in goats in the present study. Modulating immune function is one of the important functions of arginine, which is synthesized from citrulline (Zhao et al., 2018). Arginine catabolism also produces polyamines through the arginase pathway (Wu et al., 2009) that can activate toll-like receptors (TLRs) and in turn activate innate immunity (Handa et al., 2018). The essential amino acid threonine, which was also lower in the NH goats, is needed for immunoglobulin production and influences glutathione synthesis (Li et al., 2007). Leucine concentrations were lower in the NH goats compared to the H goats, while the three branched-chain amino acids, leucine, isoleucine, and valine, decreased with transportation time. These amino acids also have multiple roles, such as regulation of immunity and energy homeostasis (Coleman et al., 2020). The lower concentrations of these amino acids in goats not habituated to trailers indicate that these animals could have compromised anti-inflammatory and immune capacities, which could collectively make them more susceptible to infections after long journeys.

Methionine, which decreased with transportation time in goats, is needed for glutathione and taurine synthesis (Lushchak, 2012). Methionine could support phosphatidylcholine and carnitine by providing methyl groups to form S-adenosyl methionine (Vance et al., 1997). Lysine is one of the conserved amino acids due to its ability for slower catabolism (Flodin, 1997). Smirga et al. (2002) reported that dietary lysine deficiency increases stress-induced anxiety by enhancing serotonin release from amygdala. In our study, lysine concentrations decreased over transportation time in goats; however, serotonin concentrations did not increase with time. Lysine acts akin to a receptor competitor of serotonin and inhibits serotonin receptor-mediated anxiety, although lysine does not influence plasma serotonin concentrations (Venzi et al., 2016), which explains the pattern of serotonin concentrations over transportation time. It is not clear if these metabolites had an influence on the emotional status of goats in the present study.

### Acylcarnitines

The NH goats had significantly higher octadecanoylcarnitine (C18), hexadecanoylcarnitine (C16), tetradecenoylcarnitine (C14:1), octadecenoylcarnitine (C18:1), tetradecanoylcarnitine (C14, and decanoylcarnitine (C10) concentrations than the H goats (Table 1). Also, plasma long-chain acylcarnitines invariably increased with increasing transportation time (Supplementary Figure S3). In addition to energy expenditures due to maintaining posture and balance in a moving livestock trailer, extended transportation also imposes metabolic stress in goats since they are deprived of feed and water. The animal’s ability to cope up with metabolic stress depends on energy-production pathways, such as fatty acid oxidation in mitochondria and the TCA cycle (McCoin et al., 2015; Ghaffari et al., 2020).

The carnitine system, consisting of carnitine, acylcarnitines, carnitine enzymes and carnitine transporters, plays a crucial role in energy generation in cells (Peluso et al., 2000). The endogenous carnitine pool is comprised of the water-soluble compound l-carnitine and its esters, acylcarnitines. Although carnitine is present in highest quantity in muscle, it is mainly synthesized in the liver from the amino acids lysine and methionine and then transported through circulation. Medium and long-chain acylcarnitines that increased during transportation, particularly in the NH goats, are produced by fatty acid oxidation, while short-chain acylcarnitines are mainly synthesized from amino acids and fatty acids (Makrecka-Kuka et al., 2017). The mitochondrial carnitine system plays an indispensable role in β-oxidation of long-chain fatty acids (Calo et al., 2006). The long-chain fatty acids are transferred from the cytoplasm to the mitochondrial matrix by carnitine and acylcarnitines regulated by carnitine palmitoyltransferase 1, which is present in the mictochondrial outer membrane. After being transferred into the mictochondria, the enzyme carnitine palmitoyltransferase 2, present in the matrix, regulate regeneration of carnitine and long-chain acyl-CoA (Schooneman et al., 2013). The β-oxidation of fatty acyl CoA produces acetyl CoA, which enters the TCA cycle to generate NADH/FADH_2_ for utilization in the electron transport chain. Acylcarnitine reconversion in mitochondria is an important step that controls the amounts of fatty acids entering mitochondria from cytoplasm for β-oxidation. During times of intense workload, the acylation state of cytoplasmic carnitine pool increases more than the mitochondrial carnitine pool, suggesting that acylcarnitines are exported out of muscle cells (Ramsey and Andruini, 1993). This cellular efflux may cause increase in circulating acylcarnitine concentrations (Veld et al., 2009). Acylcarnitines in blood is the total from different tissues, as they are utilized by tissues such as skeletal muscle, cardiac muscle, and liver (Simcox et al., 2017). Increased concentrations of acylcarnitines in circulation is primarily due to muscle contraction during exercise that increases glucose and fatty acid oxidation (Hiatt et al., 1989).

Elevation in blood long-chain acylcarnitine concentrations can occur if there is a deficiency of carnitine palmitoyltransferases, incomplete β-oxidation of long-chain fatty acids, or depletion of TCA cycle intermediates (Schooneman et al., 2013; Yang et al., 2018; Ghaffari et al., 2020). Acylcarnitine concentrations can increase when the rates of β-oxidation are greater than those of the TCA cycle (Yang et al., 2019). Another reason for elevated long-chain acylcarnitine concentrations in plasma of goats during extended transportation, particularly in NH group, is elevated NEFA concentrations to meet the energy demand. The intense mobilization of fat is also reflected in elevated β-hydroxybuyrate in the NH group due to higher stress experienced during transportation than for the H group. Animal studies have indicated that approximately one-third of NEFA taken up by muscle is directly converted to long-chain acylcarnitines to meet oxidative needs (Sun et al., 2006). Xu et al. (2011) observed that high NEFA concentrations curb carnitine palmitoyltranferase-1 activity and fatty acid oxidation in cultured bovine hepatocytes. Ghaffari et al. (2019) reported that increased serum acylcarnitine concentrations coincided with increased NEFA concentrations in periparturient cows with high body condition. The increase in long-chain acylcarnitines may reflect enhanced lipolysis and the resultant β-oxidation rate that is greater than that of the TCA cycle (Ghaffari et al., 2019). The ensuing accumulation of fatty acids in the matrix could result in mitochondrial stress and incomplete fatty acid oxidation leading to acylcarnitines entering the circulation (Koves et al., 2008). In the present study, the rate of fatty acid oxidation was likely not able to cope up with the rate of accumulation of long-chain fatty acyl CoA in the mitochondrial matrix, that could have resulted in the increase in long-chain acylcarnitine concentrations in the blood.

Prolonged stress due to extended transportation can also promote inflammatory reactions in goats, particularly in those not habituated to transport trailers. Rutkowsky et al. (2014) reported that long-chain acylcarnitines activate pro-inflammatory pathways in rodent macrophages. These authors found that elevated palmitoylcarnitine (C16) concentrations increase release of interleukin-6 (IL-6) in monocytes and adenylate kinase (AK) in macrophages, the latter being a death marker. Long-chain acylcarnitines have also been reported to be associated with increased reactive oxygen species, apoptosis, and endoplasmic reticulum stress in cardiac muscle (Son et al., 2010). However, McCoin et al. (2015) did not observe markers for endoplasmic reticulum stress with increased C16 carnitine in their study, although the authors found increases in intracellular calcium and caspase-3 activity, and rapidly activated JNK/ERK/p38 MAPK stress pathways. McCoin et al. (2015) also opined that increase in long-chain acylcarnitines could promote muscle cell inflammation and stress under conditions that affect fatty acid oxidation. These reactions as a result of higher plasma long-chain acylcarnitines, if also occurring in goats, could negatively impact muscle metabolism and possibly meat quality characteristics. Conditioning goats to livestock trailers could attenuate the negative effects, such as inflammation and compromised immune function due to transportation stress. Further studies are needed to understand the potential effects of elevated plasma long-chain acylcarnitines on muscle cell inflammation, muscle metabolism, and meat quality characteristics in goats.

The beneficial effects of habituating goats to livestock trailers is clearly seen in the pattern of increase in the three long-chain acylcarnitines (C16, C18, C18:1) in the plasma (Figure 6). In the NH goats, these three long-chain acylcarnitines increased steeply with transportation time, while in the H goats, the increase was moderate, explaining the significant TRT × Time interaction effects.

Carnitine concentration was also lower in the NH goats compared to the H goats. The lower free carnitine concentrations in serum could be due to enhanced utilization by skeletal muscle fibers (Yang et al., 2019) for transferring long-chain fatty acids from cytosol into mitochondrial matrix because of intense stress and energy need. Carnitine maintains the balance between free and esterified CoA (Sharma and Black, 2009).

### Phosphatidylcholines and sphingomyelins

The phosphatidylcholines and sphingomyelins significantly affected by TRT were lower in the H goats compared to the NH goats (Table 2). In mammalian cells, the primary phospholipid that forms membranes is phosphatidylcholine, a glycerophospholipid that has a polar phosphocholine head group and two non-polar hydrocarbon chains (Taylor et al., 2007). Lyso-phosphatidylcholine is usually formed when the enzyme phospholipase 2 cleaves the fatty acid from the cell membrane phosphatidylcholine glycerol backbone. Small variations in phospholipid levels can have significant effects on lipid profiles and insulin signaling (van der Veen et al., 2017). In hepatocytes, 1-palmitoyl-2-oleoyl-*sn*-glycerol-3-phosphocholine acts as an endogenous ligand for PPATα, a transcription factor that regulates expression of multiple genes that are involved in lipid metabolism (Kersten, 2014). This nuclear hepatocyte receptor has also been reported to control a lipogenic pathway that regulates fatty acid uptake and β-oxidation by muscle (Furse and de Kroon, 2015). The elevated levels of phosphatidylcholines and lyso-phosphatidylcholines are likely associated with increased fatty acid metabolism in NH goats, as these animals experienced higher stress compared to the H goats, based on plasma NEFA concentrations.

Lyso-phosphatidylcholine (20:4) has been suggested as a marker of stress and depression in rats and humans (Adams et al., 1996; Wu et al., 2019). Although phosphatidylcholines are involved in normal cognition (Haus et al., 2009), and chronic stress can cause depression due to decreases in phosphatidylcholines (Ren et al., 2018), high levels as seen in the present experiment are due to enhanced fatty acid metabolism. Increases in plasma sphingosine and sphinganine could also result in increase in ceramide, which can cause depression (Gulbins et al., 2013).

### Other metabolites

Ketone bodies have a glucose-sparing role in ruminants and are used as a source of energy in the small intestines and peripheral tissues in ruminants (Penner et al., 2011). In the NH group of goats, β-hydroxybutyrate concentrations were higher compared to the H goats (Table 3). The metabolic precursor of β-hydroxybutyric acid, acetoacetate, is a metabolite of fatty acids (ex. butyrate) and ketogenic amino acids (ex. Leucine and isoleucine). Both butyrate and leucine concentrations were significantly lower in the NH goats in the present study (Tables 1, 3), indicating both fatty acids and amino acids were used up to a higher degree in the NH goats in producing energy during transportation. Some animal species may have a unique way of coping up with ketosis, for instance, β-hydroxybutyrate was not significantly affected as a result of 5-h transportation in dromedary camels (Wensvoort et al. (2004).

α-ketoglutarate is an important molecule that determines the rate of the TCA cycle (Wu et al., 2016). Under normal conditions, it promotes protein synthesis and curbs protein breakdown. In the present study, α-ketoglutarate was lower in the NH groups compared to the H group (Table 3), and the significant TRT × Time interaction effect noticed was because of the rapid decline of this metabolite in the H goats after loading onto the trailer (Figure 6). Decrease of this key TCA cycle intermediate could have also contributed to the possible enhanced level of β-oxidation, resulting in fatty acid overload and the consequent increase in long-chain acylcarnitines in circulation. α-ketglutarate could synthesize glutamate, which was also lower in the NH group (Table 1). α-ketoglutaric acid plays an important role in immune function of the organism. Glutamine, that is formed by glutamate, is an important fuel for lymphocytes and macrophages (Parry-Billings et al., 1990) that are part of the innate defense system. In addition, α-ketoglutarate can enhance cellular antioxidant capacity by increasing superoxide dismutase and glutathione peroxidase activities and preventing lipid peroxidation, as well as by scavenging reactive oxygen species (Velvizhi et al., 2002; Mailloux et al., 2009). The lower α-ketoglutarate levels in goats during transportation could negatively impact immune function and antioxidant activities.

α-aminoadipic acid was lower in the NH group and decreased with transportation time in goats in our study (Table 3). Under normal resting conditions, lower α-aminoadipic acid and higher lysine levels will result in protein synthesis (Goldansaz et al., 2020). α-aminoadipic acid is a breakdown product of lysine (Guidetti and Schwarcz, 2003), a ketogenic and indispensable amino acid. The decrease in plasma aminoadipic acid concentrations with transportation time corresponded with decrease in lysine concentrations in our study. This suggests that the breakdown of lysine *via* the saccharophine pathway does not stop with the formation of aminoadipic acid due to the energy demand during stress. The initial step in the catabolism of α-aminoadipic acid is transamination with α-ketoglutarate to form glutamate and then 2-ketoadipic acid. Subsequently, glutaryl-CoA is formed by decarboxylation of 2-ketadipic acid, which is then metabolized through the CoA esters to form acetyl-CoA (Matthews, 2020). The lower α-ketoglutaric acid noticed in the NH goats in the present study is likely due to the intensity of this catabolic process during extended transportation.

Tryptophan, that has both ketogenic and glucogenic properties was lower in the NH goats and decreased with transportation time in our study, which also corresponded with the decrease in kynurenine concentrations (Table 3). Kynurenine is degraded during tryptophan catabolism. Butyric acid concentrations were lower in the NH group compared to the H group (Table 3), and the concentrations also decreased over transportation time (Supplementary Figure S4). Butyric acid is produced from rumen microbial fermentation of dietary fiber, and its concentrations in blood negatively correlates with certain inflammatory markers (Juanola et al., 2019). Butyrate has also been shown to have anti-inflammatory and immune boosting capacities, in addition to its crucial role in energy homeostasis (Kasubuchi et al., 2015). The metabolite profiles together suggest the goats that have been previously conditioned to livestock trailers before a long-distance transportation may have better ability to cope with the negative effects of stress.

The limitation in this study was that other potential factors such as weather, noise, and vehicular vibration could not be evaluated separately due to the confounding nature of these stressors. The extent to which these factors influenced the metabolic profiles of individual goats is therefore not clear in this study.

## Conclusion

Habituation to trailers could be beneficial to goats in maintaining energy metabolism during transportation as glucogenic and ketogenic amino acid levels in blood were lower, and their decrease over transportation time was greater in the non-habituated goats compared to habituated goats. There is evidence in this study that both gluconeogenesis and fatty acid oxidation pathways are upregulated and that there is possible mitochondrial overload and incomplete fatty acid oxidation during prolonged intense stress, such as transportation, that results in elevated blood long-chain acylcarnitine concentrations. We suggest that long-chain acylcarntines could be good indicators of prolonged stress in goats as these metabolites increased with increasing transportation time, more so in the non-habituated goats, although further studies focusing on biomarker sensitivity and specificity are required. The potential negative effects of elevated long-chain acylcarnitines on myofiber inflammation, muscle metablolism, and meat quality characteristics also require further investigation. Habituation to trailers can also be beneficial in enhancing stress coping abilities in goats during long-distance transportation due to higher concentrations of metabolites that support energy homeostasis, antioxidant activities, and immune function.

## Data Availability

The original contributions presented in the study are included in the article/Supplementary Material, further inquiries can be directed to the corresponding author.
