# Supplementary material for: Metabolomic exploration of the effects of habituation to livestock trailer and extended transportation in goats
Source: Front Mol Biosci. 2022 Nov 17;9:1027069. doi: 10.3389/fmolb.2022.1027069 (PMC9714579; doi:10.3389/fmolb.2022.1027069)
Supplement: Supplementary file 1 [file DataSheet1.PDF]

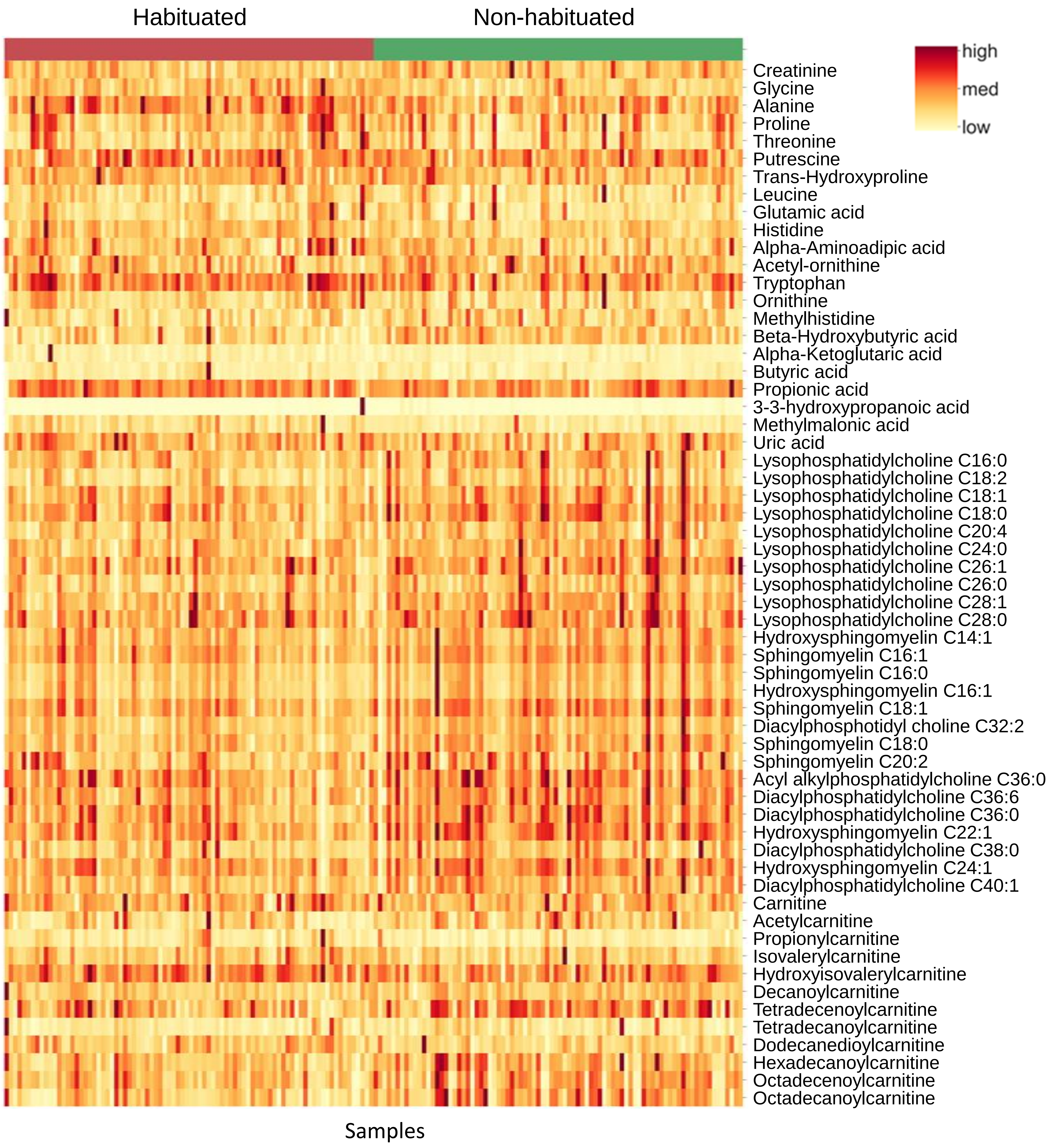

SUPPLEMENTARY FIGURE 1. Heat map of significantly affected plasma metabolites clustered by treatment (H = Habituated; NH = Non-habituated; $P < 0.05$)

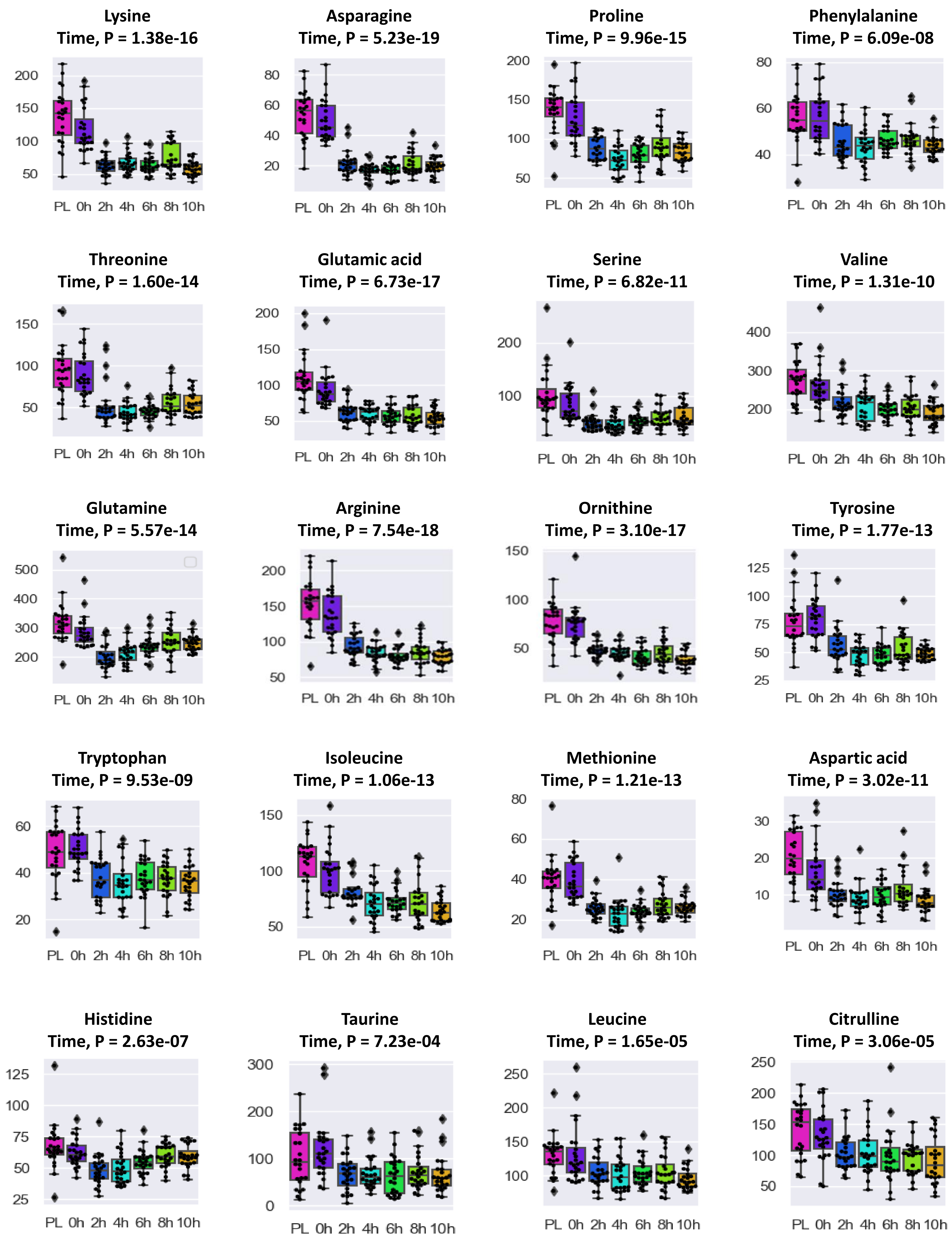

SUPPLEMENTARY FIGURE 2. Plots of amino acid concentrations (µM) in goats significantly ($P < 0.05$) affected by transportation time (Time main effect, $P < 0.05$; PL = pre-load concentrations).

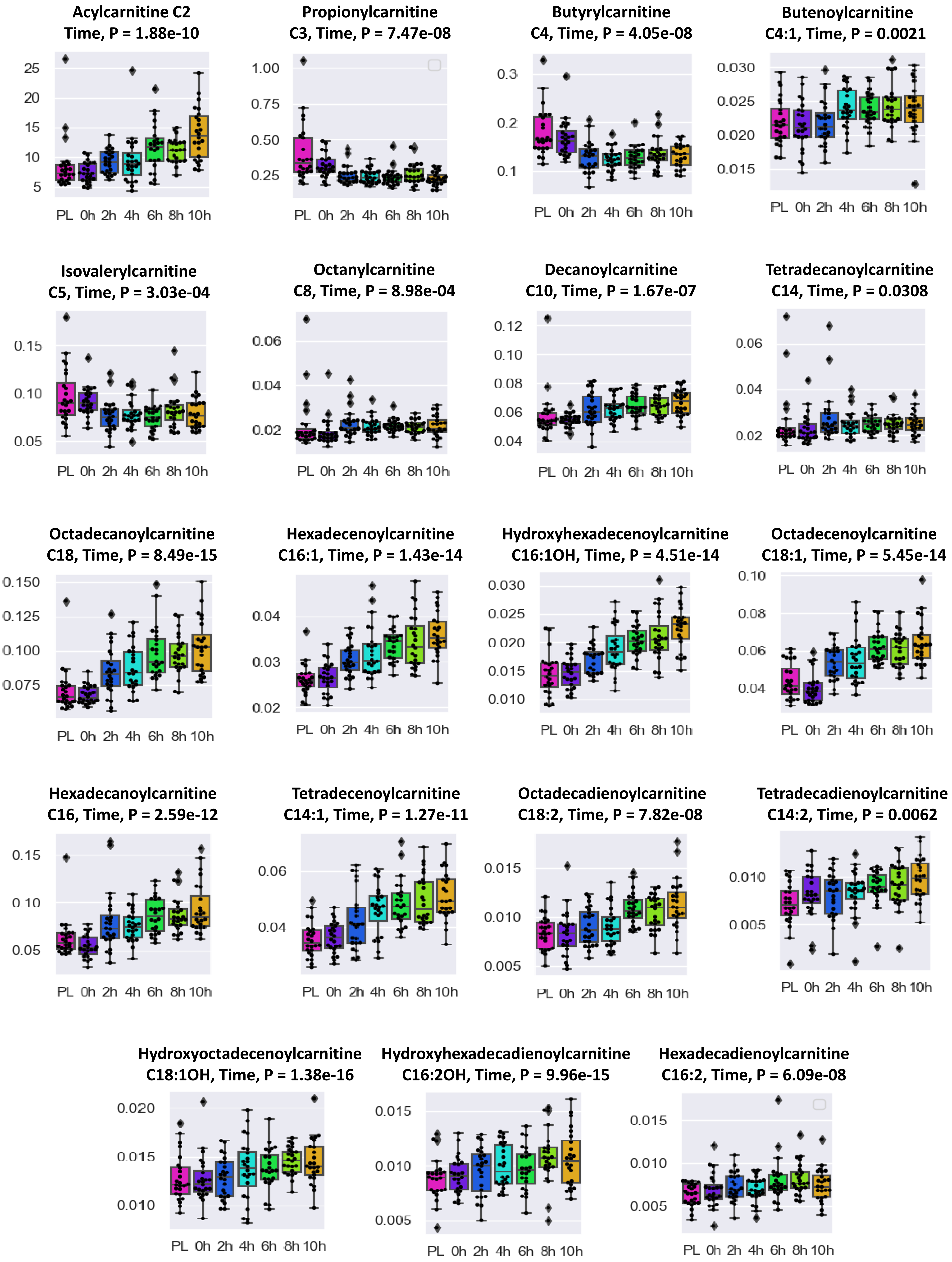

SUPPLEMENTARY FIGURE 3. Plots of acylcarnitine concentrations (μM) in goats significantly (P < 0.05) affected by transportation time (Time main effect, P < 0.05; PL = pre-load concentrations).

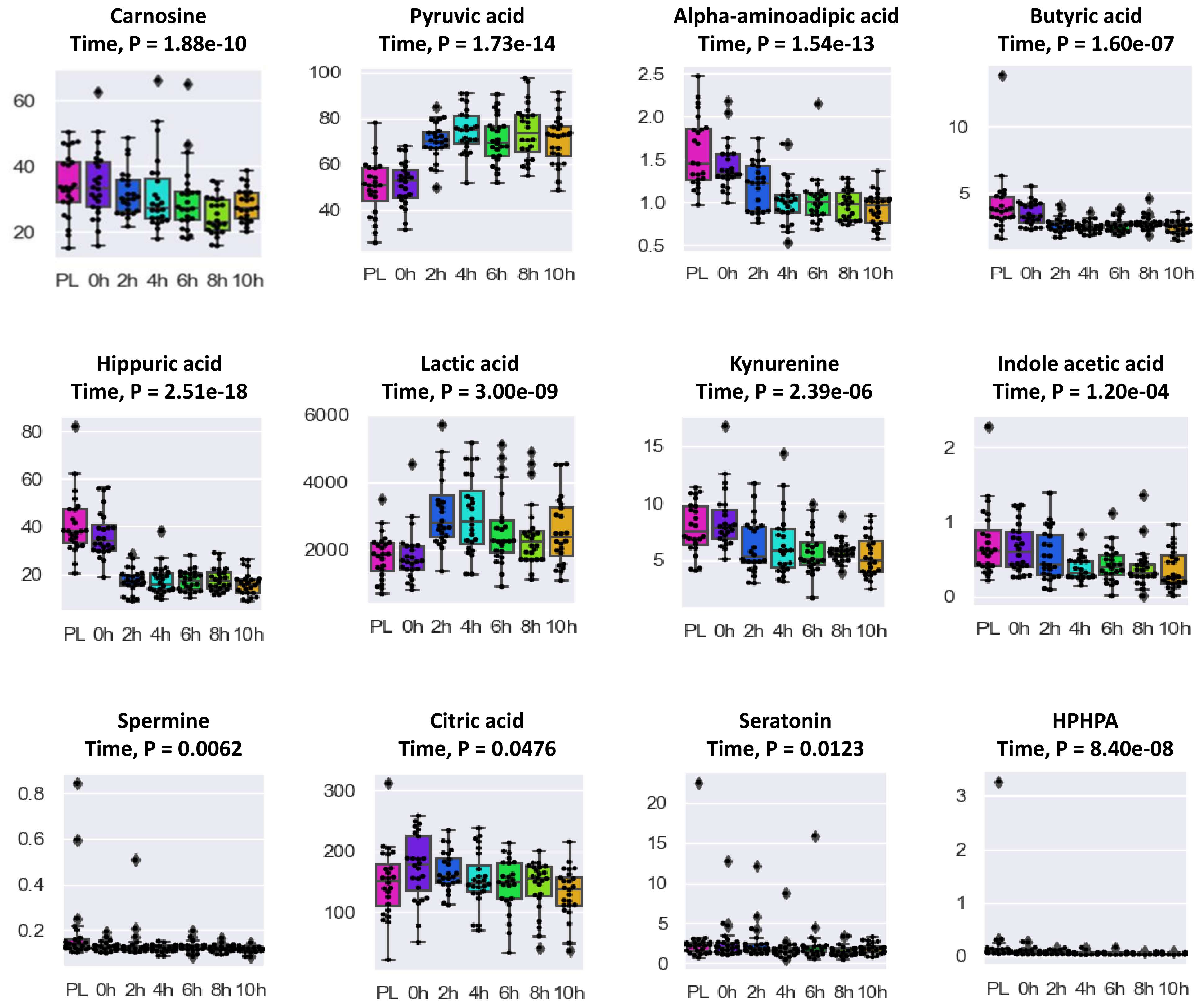

SUPPLEMENTARY FIGURE 4. Plots of metabolite concentrations ((µM) in goats significantly (P < 0.05) affected by transportation time (Time main effect, P < 0.05; PL = pre-load concentrations).

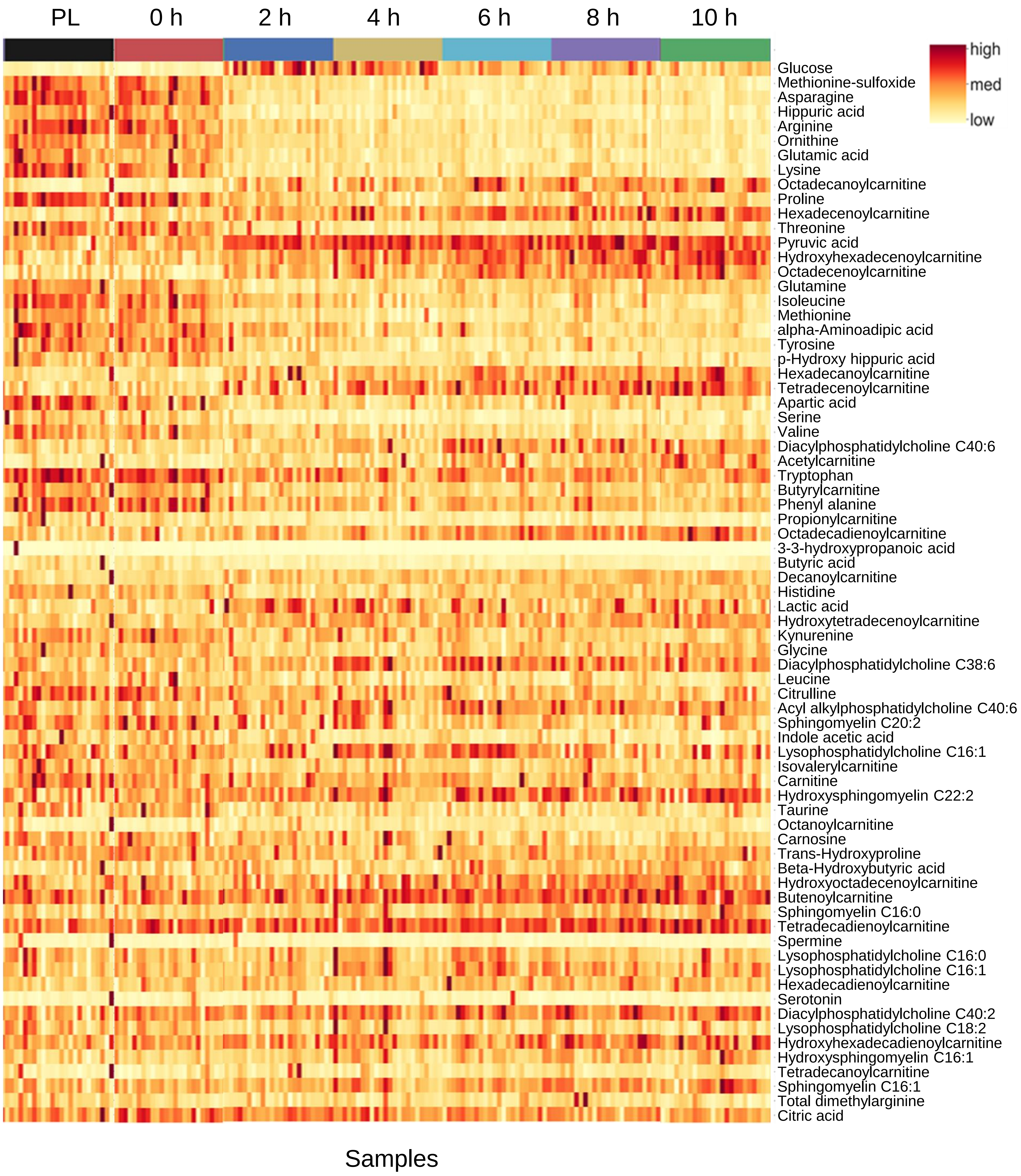

SUPPLEMENTARY FIGURE 5. Heat map of significantly affected plasma metabolites clustered by transportation time (Time; $P < 0.05$).
